# Retinal and Choroidal Vascular Changes Associated with Vitamin D Deficiency

**DOI:** 10.3390/nu18183006

**Published:** 2026-09-14

**Authors:** Tudor Corneliu Tarași, Vlad Constantin Donica, Vlad-Andrei Cianga, Dragoș-Constantin Aniță, Mircea Lazăr, Ștefan-Dragoș Tîrnovanu, Daciana Elena Brănișteanu, Anisia Iuliana Alexa, Daniel-Constantin Brănișteanu

**Affiliations:** 1 Grigore T. Popa University of Medicine and Pharmacy Iasi, 700115 Iasi, Romania; tarasi_tudor-corneliu@d.umfiasi.ro (T.C.T.); vlad-andrei.cianga@umfiasi.ro (V.-A.C.); stefan-dragos.tirnovanu@umfiasi.ro (Ș.-D.T.); daciana.branisteanu@umfiasi.ro (D.E.B.); anisia-iuliana.alexa@umfiasi.ro (A.I.A.); daniel.branisteanu@umfiasi.ro (D.-C.B.); 2 Ion Ionescu de la Brad Iasi University of Life Sciences, 700490 Iasi, Romania; dragos.anita@iuls.ro (D.-C.A.); mircea.lazar@iuls.ro (M.L.); 3 Department of Dermatology, Cai Ferate Clinical Hospital, 700506 Iasi, Romania; 4 Department of Ophthalmology, Cai Ferate Clinical Hospital, 700506 Iasi, Romania

**Keywords:** vitamin D deficiency, optical coherence tomography angiography, retinal microvasculature, choroidal vasculature, vessel density

## Abstract

Vitamin D is involved in endothelial regulation, inflammatory control, oxidative stress responses, angiogenesis, and neurovascular homeostasis, providing a biological basis for its potential involvement in the retinal and choroidal circulation. This narrative review synthesizes evidence from studies investigating associations between serum 25-hydroxyvitamin D [25(OH)D] concentrations and ocular vascular parameters assessed using optical coherence tomography (OCT), OCT angiography (OCTA), and retinal vascular imaging. Evidence from adult and pediatric populations generally associated vitamin D deficiency (VDD) with lower vessel density in the superficial and deep capillary plexuses, altered retinal vascular caliber and foveal avascular zone morphology, and reduced choroidal thickness or vascularity. However, the direction and magnitude of retinal OCTA findings were inconsistent, and conventional retinal structural measurements frequently remained unchanged. Supplementation studies reported relatively consistent increases in choroidal thickness and vascularity, whereas retinal perfusion responses were limited. Preliminary studies suggested that correction of VDD might support a more sustained response to bevacizumab in diabetic macular edema and central retinal vein occlusion, although this effect was not demonstrated consistently across retinal vascular diseases. Lower 25(OH)D concentrations were also more frequent among patients with retinal vascular occlusions, but residual systemic vascular confounding limits causal interpretation. Overall, current evidence supports a biologically plausible association between vitamin D status and ocular microvascular health but does not establish causality, functional visual benefit, or a specific therapeutic indication for supplementation. Longitudinal multicenter studies and randomized trials using standardized imaging protocols, functional outcomes, biochemical confirmation of vitamin D correction, and validated automated image analysis are required to determine the clinical relevance and reversibility of these findings.

## 1. Introduction

Vitamin D status has emerged as a potential determinant of ocular microvascular health. Total serum 25-hydroxyvitamin D [25(OH)D] represents the principal circulating marker used to assess vitamin D status [1]. Using receptor-mediated signaling, vitamin D may influence endothelial function, nitric oxide availability, inflammatory and oxidative pathways, vascular integrity and homeostasis [2]. Growing evidence linking vitamin D deficiency (VDD) to systemic endothelial and microvascular dysfunction has stimulated interest in its potential effects on the retinal and choroidal circulations. These vascular networks are particularly relevant because they support tissues with high metabolic demands and can be evaluated non-invasively using advanced ocular imaging.

The retinal and choroidal circulations have distinct but complementary physiological roles. The retinal circulation supports the metabolically active inner retinal layers, whereas the choroidal circulation supplies the retinal pigment epithelium and outer retina, including the photoreceptors [3]. The introduction of optical coherence tomography angiography (OCTA) enables non-invasive, depth-resolved visualization of the retinal and choroidal microvasculature and allows the measurement of the vessel density (VD) within the superficial and deep capillary plexuses, as well as quantitative assessment of the foveal avascular zone (FAZ). OCTA may therefore identify subtle perfusion abnormalities that are not apparent on clinical examination or conventional structural OCT [4].

Observational studies in adult and pediatric populations have reported associations between serum vitamin D status and retinal or choroidal imaging characteristics. In adults, lower 25(OH)D concentrations have been associated with differences in retinal microvascular perfusion and choroidal structure, although the direction and magnitude of these findings have varied across studies [5,6,7,8,9]. Retinal and choroidal differences have also been observed in children with VDD [10,11]. Collectively, the available evidence indicates that vitamin D status may be associated with ocular vascular characteristics across different age groups, although a consistent VDD-specific imaging phenotype has not been identified.

The possible clinical relevance of these associations has been investigated in diabetes, retinal vascular occlusion, and anti-vascular endothelial growth factor (VEGF) therapy. In patients with diabetes, lower vitamin D concentrations have been associated with more pronounced retinal microvascular abnormalities and more advanced diabetic retinopathy [9,12,13]. However, the accompanying effects of hyperglycemia, inflammation, oxidative stress, and systemic endothelial dysfunction complicate the independent interpretation of vitamin D status. Lower vitamin D concentrations have also been reported in patients with retinal vascular occlusion [14,15]. More recently, clinical interest has extended to retinal and choroidal changes following vitamin D supplementation and to the possible relationship between vitamin D correction and anti-VEGF treatment response. Evidence in these therapeutic settings remains preliminary and does not currently demonstrate an independent treatment effect.

Because the retinal microcirculation can be examined non-invasively, ocular vascular measurements may provide insight into systemic microvascular health. VDD has been associated with abnormalities in other vascular territories, including the coronary microcirculation and cerebral small vessels [16,17]. Retinal and choroidal imaging findings associated with lower vitamin D concentrations may therefore reflect a broader systemic vascular phenotype rather than an isolated ocular process. Nevertheless, a comparative analysis between ocular and systemic vascular beds requires caution because ocular perfusion is influenced by distinctive anatomical and physiological factors, including the dual retinal and choroidal blood supply, the blood–retina barrier, local autoregulation, neurovascular coupling, and intraocular pressure [18,19].

Among the nutritional and bioactive compounds investigated in relation to ocular health, vitamin D is of particular interest because it functions as a secosteroid hormone rather than solely as a dietary nutrient or antioxidant. Circulating vitamin D provides a measurable indicator of systemic vitamin D status, while its local conversion to active forms and subsequent receptor-mediated transcription allow tissue- and cell-specific responses. The presence of VDR and vitamin D-metabolizing enzymes in retinal, RPE, and choroidal tissues therefore provides a potential biological link between systemic vitamin D status and local vascular, inflammatory, angiogenic, and neurovascular regulation. Nevertheless, interpretation of available evidence is limited by substantial heterogeneity in study populations, sample sizes, eligibility criteria, definitions of VDD, OCT and OCTA devices, scan dimensions, segmentation procedures, image-processing methods, and definitions of vascular outcomes. Adjustment for potential confounders, including age, sex, body mass index, sunlight exposure, systemic disease, medication use, and ocular characteristics, has also been inconsistent. Consequently, it remains uncertain whether the reported imaging findings represent an independent association with vitamin D status, accompanying systemic conditions, or technical variation among studies, and whether VDD precedes the observed abnormalities, whether these changes are reversible following supplementation, or whether they have functional visual or therapeutic significance.

Within the extensive literature concerning vitamin D and ocular health, the added value of the present review lies in its specific focus on quantitative retinal and choroidal vascular findings obtained using OCT and OCTA and in its integration of adult and pediatric observational evidence with preclinical mechanisms, supplementation studies, anti-VEGF therapy, and retinal vascular occlusive disease. Accordingly, this narrative review aims to critically synthesize the evidence concerning vitamin D status and retinal and choroidal vascular characteristics, compare findings across adult and pediatric populations, examine imaging changes following vitamin D supplementation, evaluate the relationship of vitamin D status and its correction with anti-VEGF treatment outcomes and retinal vascular occlusive disease and identify methodological priorities for future research. This focused approach is intended to distinguish biological plausibility from demonstrated clinical association and from currently unproven therapeutic benefit.

## 2. Materials and Methods

The literature search was primarily conducted in PubMed using a structured search approach. PubMed was searched on 5 July 2026 for studies published during the preceding five years. The search used combinations of the following terms with the Boolean operators AND and OR: “vitamin D,” “retina,” “retinal,” “vascular,” “optical coherence tomography,” “optical coherence tomography angiography,” “OCT,” and “OCTA.” Separate searches were performed using different combinations of these terms according to the topic being investigated. Supplementary targeted searches were subsequently performed in Scopus and ScienceDirect to identify additional potentially relevant studies and to complement the primary PubMed search. These supplementary searches used combinations of the same core concepts related to vitamin D, retinal and choroidal vascularization, and OCT/OCTA imaging; however, the combinations and filters were adapted according to the specific topic being explored rather than applying an identical predefined search strategy in all databases. The database-specific search approach and principal search terms are provided in Appendix A.

Original human studies were eligible if they evaluated the relationship between vitamin D status and the retinal or choroidal circulation. Eligible outcomes included retinal and choroidal microvascular parameters assessed using OCT or OCTA, fundus-based retinal vascular measurements, retinal vascular occlusive diseases, vascular changes following vitamin D supplementation, and the influence of VDD or its correction on the response to anti-VEGF therapy. Cross-sectional, case–control, cohort, and interventional studies involving adult or pediatric populations were considered.

Studies were excluded if they were published outside the predefined period, were not written in English, did not involve human participants, did not assess vitamin D status or supplementation, or did not report outcomes relevant to the retinal or choroidal circulation. Reviews, meta-analyses, conference abstracts, editorials, letters, protocols, preprints, animal studies, and in vitro investigations were also excluded.

The database search identified 125 records. After the removal of 31 duplicates, 94 unique records underwent title, abstract, and eligibility screening. Of these, 23 studies met the inclusion criteria and were included in the narrative synthesis; the remaining 71 records were excluded.

**Figure 1:**
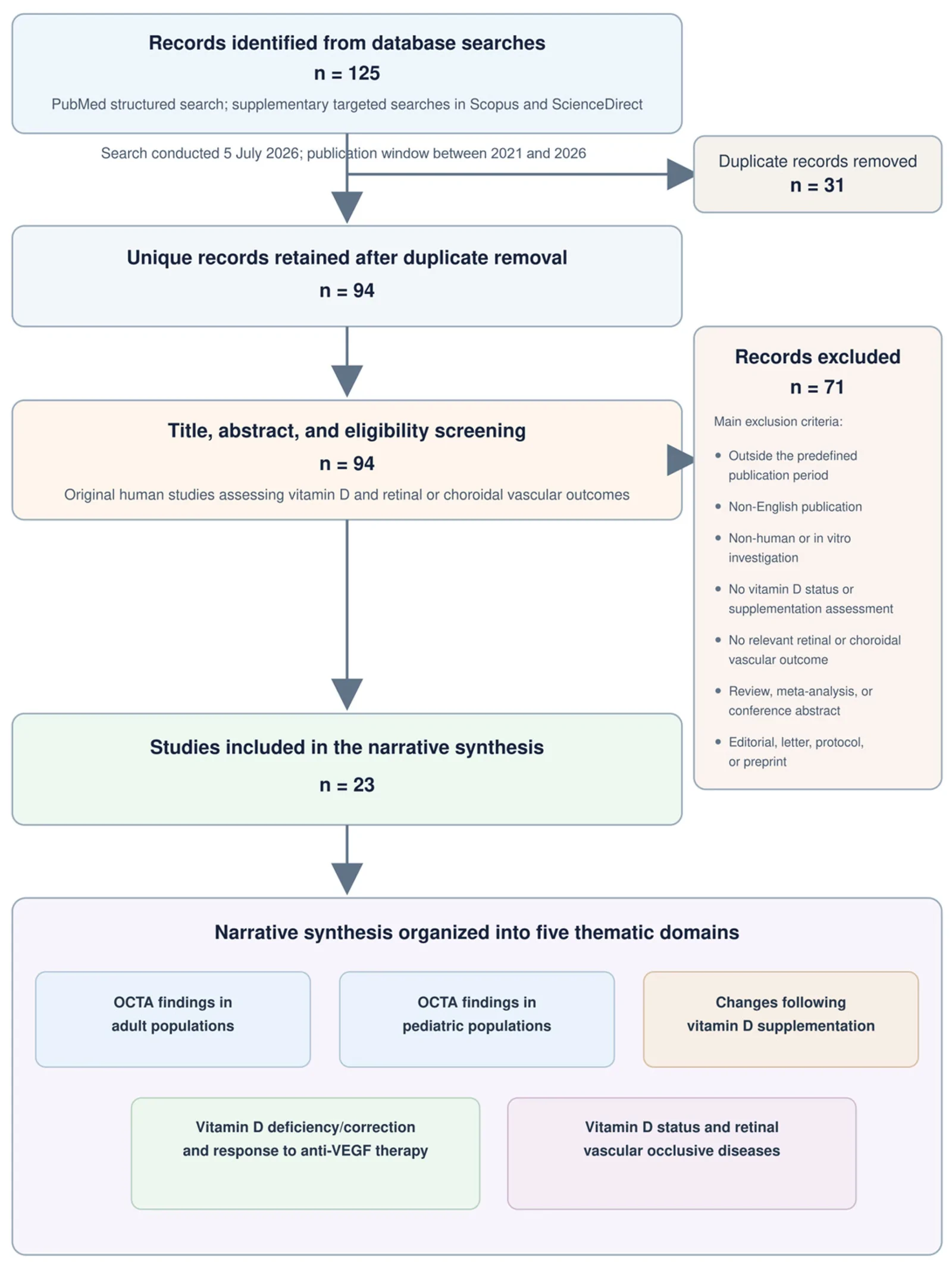
Study identification and selection process.

For each included study, data were extracted regarding the study population and sample size, participants’ age, serum vitamin D levels, investigated retinal or choroidal parameters, principal results, and key aspects relevant to interpreting the findings. The evidence was narratively synthesized and organized into five thematic domains: OCTA findings in adult populations, OCTA findings in pediatric populations, vascular changes following vitamin D supplementation, the influence of VDD and its correction on the response to anti-VEGF therapy, and the association between vitamin D status and retinal vascular occlusive diseases. Data are presented as mean ± standard deviation unless otherwise indicated. Owing to the clinical and methodological heterogeneity of the included studies, a quantitative meta-analysis was not performed. A flowchart describing the search is presented in Figure 1.

To enhance methodological transparency and reporting quality, the present narrative review was appraised using the Scale for the Assessment of Narrative Review Articles (SANRA). The appraisal considered the six SANRA domains: justification of the review’s importance, statement of aims, description of the literature search, referencing, scientific reasoning, and appropriate presentation of relevant data. The item-level SANRA appraisal is provided in Appendix B.

Generative artificial intelligence tools were used exclusively for language editing, including rephrasing and improving the manuscript’s grammar, clarity, readability, and presentation. All scientific content, interpretation of findings, and conclusions were developed and critically reviewed by the authors, who retain full responsibility for the final manuscript. These tools were not used to generate research data, conduct analyses, draw conclusions, or substitute for the authors’ scientific judgment.

## 3. Vitamin D Signaling and Ocular Microvascular Homeostasis

**Figure 2:**
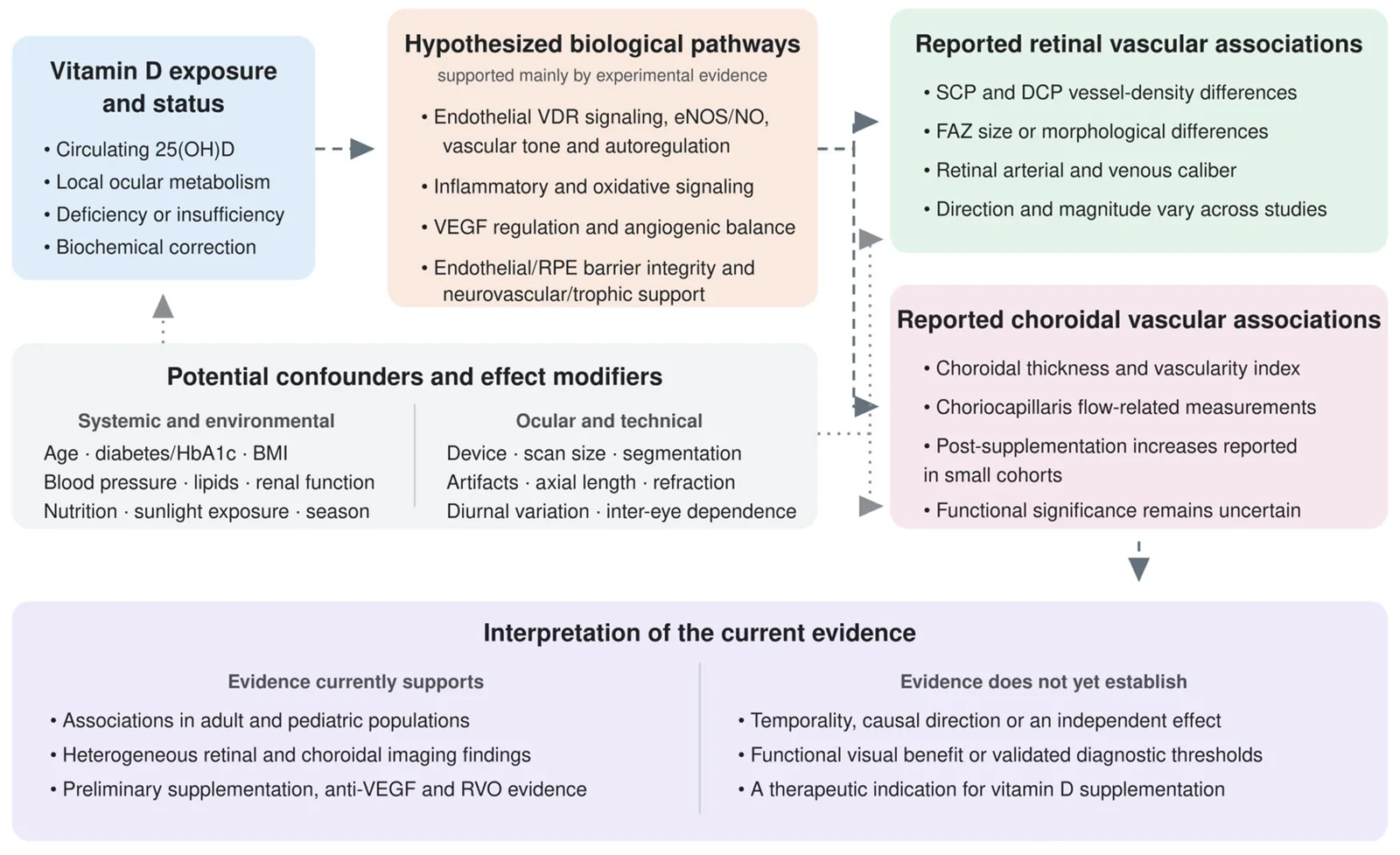
Proposed relationships between vitamin D status and retinal and choroidal vascular findings.

The association between VDD and retinal or choroidal microvascular abnormalities is biologically plausible because vitamin D signaling influences endothelial homeostasis, inflammatory activity, oxidative stress, angiogenesis, and neurovascular regulation [20,21,22]. The retina also possesses a local vitamin D metabolic system, suggesting that ocular tissues may respond both to circulating vitamin D availability and to locally produced active metabolites [23,24]. Although these mechanisms do not establish a causal relationship between VDD and OCTA abnormalities, they provide a framework for interpreting the vascular findings reported in clinical studies. Figure 2 presents a proposed relationship between vitamin D levels and ocular findings.

### 3.1. Vitamin D Metabolism and Ocular Bioavailability

Vitamin D is obtained predominantly through cutaneous synthesis following exposure to ultraviolet B radiation, while dietary vitamin D_2_ and D_3_ provide a smaller contribution [25,26]. After entering the circulation, vitamin D is transported by vitamin D-binding protein to the liver, where CYP2R1 converts it into 25-hydroxyvitamin D [25(OH)D], or calcidiol. Because of its relatively long circulating half-life, 25(OH)D is considered the most reliable biochemical marker of vitamin D. The biologically active metabolite, 1,25-dihydroxyvitamin D_3_ or calcitriol, is produced predominantly in the kidney through CYP27B1-mediated hydroxylation of 25(OH)D [27,28]. The eye possesses the enzymatic machinery required for local vitamin D metabolism. CYP27B1 converts 25(OH)D into calcitriol, whereas CYP24A1 initiates the catabolism of both 25(OH)D and calcitriol; both enzymes have been localized in the ciliary body, retinal pigment epithelium, and neural retina. Local calcitriol production may become particularly relevant under metabolic stress, including hyperglycemia in diabetes [23,29]. Differences in aqueous humor 25(OH)D concentrations between patients with and without glaucoma, even in the absence of detectable structural retinal changes, further suggest that intraocular vitamin D availability may not be fully represented by serum measurements [30].

According to one commonly used classification, serum concentrations below 25 nmol/L indicate deficiency, whereas concentrations between 25 and 59 nmol/L indicate insufficiency [31]. However, the thresholds used in ophthalmological studies vary, with some defining VDD at ≤20 ng/mL and others using values of ≤30 ng/mL. This variation may influence the magnitude and clinical interpretation of the reported vascular associations.

Systemic factors may nevertheless influence the amount of vitamin D available to ocular tissues. Reduced cutaneous synthesis, limited sunlight exposure, and declining renal hydroxylation capacity may contribute to lower vitamin D availability in older adults [28]. Disruption at any stage of vitamin D production or activation can therefore reduce both circulating 25(OH)D and the substrate available for local ocular metabolism.

### 3.2. Vitamin D Receptor Signaling

At the molecular level, 1,25-dihydroxyvitamin D_3_ [1,25(OH)_2_D_3_] binds the vitamin D receptor (VDR), a ligand-activated transcription factor belonging to the nuclear receptor superfamily. Ligand binding induces a conformational change that promotes or stabilizes the interaction of VDR with the retinoid X receptor (RXR). The VDR–RXR complex binds vitamin D response elements, with direct-repeat sequences separated by three nucleotides representing the best-characterized binding configuration. Genome-wide studies have shown that VDR-binding sites occur not only in gene promoters but also in intronic and distal regulatory regions. DNA-bound VDR recruits coactivators, the Mediator complex, and chromatin-modifying or remodeling proteins, facilitating RNA polymerase II-dependent transcription. Conversely, VDR may associate with corepressors and histone deacetylases in ligand-free or transcriptionally repressive states. Consequently, the transcriptional effects of VDR activation depend on chromatin accessibility, co-regulator availability, and cellular context [32,33,34,35].

Experimental studies have demonstrated VDR expression in retinal endothelial cells, supporting the presence of functional VDR signaling within the retinal microvasculature [21]. The cellular consequences of altered endothelial VDR expression are discussed in the following sections. Rapid, non-genomic responses to 1,25(OH)_2_D_3_ have also been described and may involve membrane-associated VDR and intracellular kinase or calcium-signaling pathways. However, these responses remain less clearly characterized than classical VDR-mediated transcription, particularly in ocular tissues [33,34].

### 3.3. Endothelial Dysfunction, Inflammation, and Oxidative Stress

Retinal endothelial cells form the inner blood–retina barrier and regulate vascular tone, leukocyte trafficking, and local inflammatory activity. Calcitriol–VDR signaling supports endothelial homeostasis by maintaining endothelial nitric oxide synthase expression and nitric oxide bioavailability. Correspondingly, VDR-deficient mouse retinal endothelial cells exhibit reduced endothelial nitric oxide synthase expression and increased inflammatory and oxidative-stress responses [21,36]. Because nitric oxide mediates basal vascular tone and metabolically stimulated vasodilation, impaired vitamin D signaling could limit retinal microvascular adaptation. Clinical associations between VDD and reduced retinal VD are compatible with this mechanism but do not establish causality.

Calcitriol may also suppress excessive renin–angiotensin system activity, thereby limiting angiotensin II-mediated vasoconstriction. Systemic associations of low 25(OH)D concentrations with impaired flow-mediated dilation and increased arterial stiffness further support a relationship between vitamin D status and endothelial dysfunction [37]. Severe deficiency has additionally been associated with prothrombotic changes that may aggravate perfusion impairment under ischemic or metabolic stress [38].

Vitamin D may attenuate inflammation by inhibiting nuclear factor kappa-B signaling and reducing mediators such as interleukin-6 and tumor necrosis factor-α [39]. Inadequate signaling may therefore promote endothelial activation, leukocyte recruitment, and increased vascular permeability. Upregulation of intercellular adhesion molecule-1 and vascular cell adhesion molecule-1 may further enhance leukocyte–endothelial interactions and slow capillary flow [21], while cytokine-driven VEGF production and dysregulated matrix metalloproteinase activity may intensify retinal microvascular injury [40]. At the same time, impaired vitamin D signaling may weaken mitochondrial and antioxidant defenses, increasing reactive oxygen species production and endothelial apoptosis, particularly during hyperglycemic stress [41]. Experimental findings in RPE cells similarly suggest that calcitriol reduces oxidative injury and preserves mitochondrial function [42,43]. Collectively, endothelial dysfunction, inflammation, and oxidative stress provide complementary pathways through which VDD could contribute to retinal microvascular impairment.

### 3.4. Vitamin D–Mediated Angiogenic and Neurovascular Regulation

Vitamin D appears to regulate angiogenesis rather than exerting a uniformly anti-angiogenic effect. Calcitriol may limit excessive VEGF expression by modulating hypoxia-inducible factor-1α signaling, while VDR activation has been shown to suppress pathological retinal neovascularization [44]. Similar effects in retinoblastoma and tumor models support a broader role in restricting uncontrolled vascular proliferation [45,46]. Inhibition of transforming growth factor-β-mediated VEGF secretion may provide an additional mechanism in ischemia-prone retinal conditions [47].

VDR expression in retinal ganglion cells and inner nuclear layer neurons suggests that vitamin D may also influence neurovascular coupling. Disrupted signaling among neurons, glial cells, pericytes, and endothelial cells could produce perfusion abnormalities before structural retinal thinning becomes detectable. Beyond its vascular effects, vitamin D signaling may contribute to retinal neuroprotection. VDR expression has been reported in the RPE and retinal neurons, including photoreceptors and retinal ganglion cells, providing a molecular basis for direct vitamin D responsiveness in these tissues [24]. In glaucomatous mice, treatment with 1,25(OH)_2_D_3_ reduced retinal ganglion cell loss and increased retinal expression of brain-derived neurotrophic factor, VEGF-A, and placental growth factor, suggesting that vitamin D signaling may support neuronal survival through trophic and anti-inflammatory mechanisms [48]. Complementary in vitro evidence indicates that vitamin D_3_ can reduce oxidative-stress-induced apoptosis in ARPE-19 cells, potentially preserving the supportive and barrier functions of the RPE [49]. Persistent VDD could weaken this adaptive signaling and reduce endothelial responses to changes in oxygen availability, perfusion, and metabolic demand.

These preclinical findings provide biological plausibility for retinal neuroprotection through trophic, antioxidant, and anti-inflammatory pathways; however, they do not establish that vitamin D supplementation prevents retinal neurodegeneration or improves visual function in humans.

In vitro studies suggest that vitamin D treatment may preserve tight-junction and epithelial-integrity markers in RPE cells [47,50]. Because the RPE normally forms the outer blood–retina barrier and mediates nutrient and waste exchange between the choriocapillaris and photoreceptors, preservation of RPE integrity could indirectly support outer-retinal metabolic homeostasis [51,52]. VDD has been associated with subretinal fibrosis in advanced AMD, although the available cross-sectional evidence does not establish causality [53]. The presence of VDR, CYP27B1, and CYP24A1 in normal choroidal melanocytes and stromal fibroblasts provides a basis for local vitamin D signaling within the choroid [54].

## 4. OCTA Manifestations of Vitamin D Deficiency

OCTA detects blood-flow signals rather than histological capillary loss. Reduced VD may therefore reflect fixed capillary dropout, reversible hypoperfusion below the device’s detection threshold, or both. Moreover, endothelial barrier dysfunction and impaired vascular regulation may precede detectable OCTA or structural changes. Subclinical microvascular dysfunction may consequently be present despite normal retinal thickness and an unremarkable routine ophthalmological examination. Reports of modest macular thinning in adults with low serum 25(OH)D suggest that structural abnormalities may become more apparent with prolonged or advanced dysfunction [55,56].

The previously described mechanisms may help explain the OCTA findings reported in individuals with VDD. Reduced nitric oxide bioavailability and increased vasoconstrictor activity may decrease capillary perfusion, whereas inflammation, leukocyte adhesion, oxidative stress, and endothelial apoptosis may contribute to capillary rarefaction. On OCTA, these processes could appear as reduced VD in the SCP or DCP, enlargement or irregularity of the FAZ, and altered choriocapillaris perfusion or choroidal vascularity. Importantly, OCTA VD values obtained using different commercial devices should not be directly compared or numerically pooled because of differences in proprietary segmentation algorithms, image-processing methods, and scan dimensions. The principal characteristics and findings of the included adult studies are summarized in Table 1.

### 4.1. Retinal Microvascular Findings

The predominant OCTA finding associated with VDD was reduced retinal capillary perfusion. Lower vessel or perfusion density in the SCP and DCP was reported in otherwise healthy adults, patients with diabetes without clinically apparent diabetic retinopathy, and individuals with established diabetic retinal disease [6,8,9,56,57]. This convergence across clinically different populations suggests that the association between vitamin D status and retinal microvascular parameters is not restricted to advanced retinal disease.

The DCP appeared particularly susceptible in several studies, with lower vitamin D concentrations associated with more extensive or stronger perfusion abnormalities in the deep retinal circulation. This pattern may reflect the anatomical complexity and relatively high metabolic demands of the deeper retinal layers. Regional analyses also suggested that parafoveal, perifoveal and temporal sectors may be especially sensitive to vitamin D status. Nevertheless, the affected regions were not uniform across studies, limiting the identification of a single anatomical biomarker of VDD-associated microvascular dysfunction.

Changes in FAZ parameters were less consistent than VD findings. Some studies reported a larger FAZ or an inverse association between serum vitamin D concentration and FAZ area, particularly in diabetic populations [6,8,9]. Other investigations found no significant differences in FAZ size or morphology, despite detecting alterations in retinal perfusion [7,56,57]. Consequently, FAZ enlargement may accompany reduced capillary perfusion in some populations, but the current evidence does not support its use as a universal marker of VDD.

An important exception to the predominant pattern was reported by Karabulut et al., who identified increased DCP VD in adults with VDD [7]. This finding may represent compensatory vascular recruitment, altered flow above the OCTA detection threshold or methodological variation rather than improved microvascular function.

Associations were comparatively consistent in diabetic populations, in which lower vitamin D concentrations were generally related to reduced SCP or DCP perfusion. Such relationships were observed both before the development of clinically detectable diabetic retinopathy and in patients with non-proliferative disease or diabetic macular edema [8,9,57]. However, diabetes independently produces endothelial dysfunction, capillary nonperfusion and neurovascular injury. The observed abnormalities may therefore reflect an interaction between vitamin D status and the diabetic microvascular environment rather than an isolated effect of VDD.

### 4.2. Retinal and Choroidal Structural Findings

Structural retinal findings were generally less consistent and less extensive than OCTA-derived perfusion abnormalities. Some studies identified localized reductions in foveal, RNFL, ganglion cell complex or ganglion cell–inner plexiform layer thickness in adults with lower vitamin D concentrations [56,58]. Conversely, retinal thickness remained relatively preserved or was not independently associated with vitamin D status in other cohorts, despite significant differences in retinal VD or FAZ measurements [8,59].

These findings suggest that structural and microvascular abnormalities do not necessarily develop concurrently. OCTA may detect differences in perfusion when conventional thickness measurements remain within normal limits, although the cross-sectional nature of the available studies does not establish that vascular abnormalities precede structural damage. Moreover, reduced OCTA VD represents reduced detectable flow and cannot distinguish reversible hypoperfusion from fixed capillary loss.

Choroidal findings showed a comparatively consistent relationship with vitamin D status. Lower serum vitamin D concentrations were associated with reduced subfoveal and regional CT in several adult populations [5,60]. A reduction in the CVI was also reported, suggesting that vitamin D status may be related not only to total CT but also to the relative contribution of the vascular luminal compartment.

The CVI may provide complementary information because CT is influenced by vascular, stromal and physiological factors, whereas CVI estimates the proportion of vascular luminal area within the total choroidal area. Nevertheless, evidence concerning choroidal involvement remains based primarily on structural OCT and image binarization. Direct evidence that VDD alters choriocapillaris perfusion remains limited.

Lower vitamin D concentrations and reduced CT have also been observed in patients with age-related macular degeneration, particularly in association with advanced disease and subretinal fibrosis [59]. These findings should be interpreted cautiously because AMD itself produces substantial changes in the RPE, outer retina and choroid. The independent contribution of vitamin D status cannot therefore be separated completely from disease severity, age and associated systemic factors.

The available evidence therefore supports an association rather than a causal relationship between vitamin D status and retinal or choroidal abnormalities. OCTA appears capable of identifying subtle microvascular differences associated with low vitamin D concentrations, but the clinical and prognostic significance of these findings remains uncertain. Longitudinal and interventional studies using standardized vitamin D thresholds and OCTA protocols are required to determine whether the observed abnormalities are reversible, predict subsequent structural damage or represent an independent consequence of persistent VDD.

## 5. Vitamin D Status and OCTA Findings in Pediatric Populations

Pediatric studies suggest that VDD may be associated with retinal and choroidal vascular alterations before clinically apparent ocular disease develops. However, the available evidence is limited and includes different imaging methods and populations. While some studies evaluated otherwise healthy children, others included children with type 1 diabetes or malnutrition. The characteristics and numerical findings of these studies are summarized in Table 2.

In otherwise healthy pediatric populations, the available findings suggest that retinal vascular measurements may be associated with vitamin D status. Lower vitamin D concentrations have been linked to narrower retinal arterioles and wider retinal venules, while OCTA-based observations have shown reduced VD in the SCP and a more localized reduction in the foveal DCP [10,11]. Taken together, these findings suggest that VDD may be associated with changes at both the retinal vessel-caliber and capillary-perfusion levels.

Choroidal findings varied according to the population and the parameter examined. In otherwise healthy children, VDD was associated with reduced CT, and vitamin D concentrations correlated positively with measurements obtained at some choroidal locations [10]. This finding is compatible with a relationship between vitamin D status and choroidal structure, although the cross-sectional design cannot determine whether low vitamin D contributes directly to choroidal thinning.

The relationship was less specific in children with type 1 diabetes. CT was lower in diabetic children than in healthy controls irrespective of vitamin D status. Although vitamin D concentration correlated positively with CT within the deficient diabetic group, choroidal measurements did not differ significantly between diabetic children with and without VDD [62]. These results suggest that diabetes may be a more important determinant of choroidal thinning in this population, while vitamin D status may act as a modifying or accompanying factor. They do not establish an independent effect of VDD on the choroid in diabetic patients.

In malnourished children, the CVI was reduced despite the absence of a significant difference in total CT. Vitamin D levels correlated positively with the CVI, raising the possibility that choroidal vascular composition may be more sensitive than overall thickness to systemic nutritional disturbances [61]. Nevertheless, malnutrition involves multiple interrelated factors, including iron status, anemia, energy deficiency, and other micronutrient deficiencies. The independent contribution of vitamin D therefore cannot be isolated from this study.

Collectively, these observations suggest that pediatric VDD may be associated with choroidal structural or vascular differences, but the direction and specificity of the association are not uniform. The pediatric analysis provides preliminary evidence linking lower vitamin D concentrations with altered retinal vascular caliber, reduced superficial macular perfusion, and differences in CT or vascular anatomy. Vascular findings appear more consistent than conventional retinal and retinal nerve fiber layer thickness measurements. Nevertheless, the evidence is derived from a small number of heterogeneous studies, and only one investigation primarily assessed the retinal microcirculation using OCTA.

## 6. OCTA Changes Following Vitamin D Supplementation

Interventional evidence regarding ocular changes following correction of VDD remains limited. Although choroidal measurements generally increased following vitamin D supplementation, retinal OCTA parameters were predominantly unchanged or showed small reductions of uncertain significance.

Post-supplementation retinal OCTA findings have been inconsistent, whereas increases in certain choroidal measurements have been reported more consistently. This difference may reflect the distinct vascular regulation, tissue composition, and metabolic requirements of the retinal and choroidal circulations. In both adult and pediatric populations, VDD was associated with lower baseline choroidal measurements, followed by increases after vitamin D replacement [63,64]. In adults, CT increased at subfoveal, nasal, and temporal locations, and serum vitamin D levels correlated positively with CT. These observations indicate that CT is responsive to changes in vitamin D status, although they do not establish that the initial thinning was caused exclusively by VDD. Supplementation protocols, study populations, follow-up intervals, and imaging outcomes are summarized in Table 3.

The pediatric study extended these findings beyond CT. Before treatment, children with VDD had lower CT, total choroidal area, luminal area, stromal area, and CVI than controls. These parameters increased following supplementation, but the extent of the response differed according to deficiency severity. Children with the most severe VDD demonstrated changes across both thickness and vascularity measurements, whereas children with milder deficiency showed changes predominantly in choroidal area and vascularity parameters, with limited changes in CT [63]. This pattern suggests that the vascular and stromal composition of the choroid may respond to vitamin D correction even when changes in total thickness are less apparent.

In apparently healthy adults with VDD, Karabulut et al. observed a reduction in DCP VD following supplementation, affecting the whole, parafoveal, and perifoveal regions. No comparable longitudinal changes occurred in the untreated group, whereas SCP VD, FAZ area, and choriocapillaris flow area remained largely unchanged [65].

Limited retinal microvascular changes were also reported following repletion of VDD in patients with type 2 diabetes without diabetic retinopathy or with mild-to-moderate non-proliferative diabetic retinopathy. Almost all OCTA perfusion and FAZ parameters remained unchanged after three months. The only significant finding was a small reduction in superior parafoveal superficial VD, which was not accompanied by similar changes in other quadrants. Moreover, changes in serum vitamin D concentration were not associated with changes in OCTA parameters or visual acuity [66].

The mechanisms underlying the reported retinal and choroidal changes after vitamin D supplementation remain uncertain. Potential explanations include VDR-mediated regulation of endothelial function, inflammatory and oxidative pathways, angiogenic signaling, and RPE or choroidal homeostasis, as discussed in Section 3. However, supplementation studies do not establish that these mechanisms account for the observed imaging changes.

## 7. Vitamin D in Anti-VEGF Therapy

Clinical evidence regarding vitamin D status and anti-VEGF treatment remains limited to a small number of studies involving intravitreal bevacizumab. These studies evaluated macular edema associated with diabetic retinopathy or retinal vein occlusion and differed in follow-up duration and disease characteristics. Overall, VDD did not prevent an initial anatomical or functional response to bevacizumab. However, uncorrected deficiency was associated with less sustained improvement in one diabetic macular edema study and with poorer outcomes in a small subgroup of patients with central retinal vein occlusion. The study characteristics and numerical outcomes are summarized in Table 4.

In DME, visual acuity and CMT improved initially irrespective of vitamin D status. Karimi et al. found no significant short-term differences between vitamin D-sufficient, supplemented, and untreated deficient patients, although CMT reduction was numerically greater following supplementation [69]. With longer follow-up, Fekri et al. reported smaller improvements in CDVA and CMT at six months among patients with uncorrected VDD. Supplemented patients achieved outcomes comparable to those with sufficient vitamin D, while injection numbers did not differ among groups [67]. These findings suggest a possible influence on response durability rather than on the initial effect of bevacizumab.

A disease-specific pattern was observed in macular edema secondary to retinal vein occlusion. Anatomical and functional improvements occurred across all vitamin D groups, and baseline vitamin D concentration was not correlated with BCVA or CMT. No supplementation-related benefit was identified in BRVO. In CRVO, however, untreated deficient patients showed smaller improvements than vitamin D-sufficient and supplemented patients [68]. This finding should be interpreted cautiously because the CRVO analysis included only 27 eyes, and biochemical correction was not confirmed after supplementation.

Available studies suggest that vitamin D correction may be associated with a more sustained bevacizumab response in DME and possibly in CRVO-associated macular edema. Potential explanations include VDR-mediated modulation of VEGF expression, inflammatory and oxidative pathways, endothelial barrier function, and tissue remodeling, as discussed in Section 3; however, these mechanisms remain unconfirmed in the clinical setting. The evidence is limited by small samples, short follow-up periods, subgroup analyses, and the exclusive use of bevacizumab and is therefore insufficient to establish vitamin D status as an independent modifier of anti-VEGF efficacy. Vitamin D supplementation should not currently be considered an established adjunct to anti-VEGF therapy. Larger randomized studies involving different anti-VEGF agents are needed to determine whether vitamin D correction influences anatomical outcomes, visual improvement, treatment durability, or injection requirements.

## 8. Vitamin D Status and Retinal Vascular Occlusions

Observational studies have evaluated the relationship between systemic vitamin D status and clinically manifest retinal vascular occlusion rather than subclinical perfusion abnormalities. Their populations, vitamin D concentrations, and principal findings are summarized in Table 5.

Studies consistently report lower serum vitamin D concentrations in patients with retinal vascular occlusions than in unaffected controls. Kandambeth et al. found that all patients with RVO had vitamin D concentrations below 30 ng/mL, while Bhattacharya et al. similarly reported significantly lower concentrations in RVO cases. Neither study identified significant differences between CRVO and BRVO, suggesting that the association is not specific to the anatomical subtype of venous occlusion [70,72].

Bhanot et al. extended these observations to both retinal venous and arterial occlusions. Vitamin D concentrations were lower in affected patients than in controls and, when all occlusion types were pooled, were lower in ischemic than in non-ischemic presentations. However, ischemic–non-ischemic differences within individual occlusion subtypes were not significant, probably because of the small subgroup sizes. Differences in macular thickness and visual acuity primarily reflected the consequences of established vascular occlusion rather than a direct effect of vitamin D [71].

Overall, low vitamin D status appears to be associated with retinal vascular occlusive disease, but causality remains unproven. Hypertension, dyslipidemia, diabetes, nutritional status, lifestyle, and sunlight exposure may confound this relationship. Consequently, VDD should be corrected for established systemic indications, but current evidence does not support vitamin D supplementation as a targeted treatment or preventive intervention for retinal vascular occlusion.

## 9. Limitations and Clinical Implications

### 9.1. Limitations

Several limitations should be considered when interpreting the evidence synthesized in this review. Most included studies used cross-sectional or case–control designs, precluding conclusions regarding temporality and causality. It therefore remains uncertain whether VDD directly contributes to retinal and choroidal vascular impairment, increases susceptibility to pre-existing disease, or reflects broader systemic endothelial and metabolic dysfunction. Supplementation studies provide preliminary evidence of ocular responsiveness to vitamin D correction; however, their small samples, short follow-up periods, heterogeneous treatment regimens, and inconsistent biochemical confirmation limit conclusions regarding reversibility or therapeutic efficacy.

The study populations were heterogeneous and included healthy children and adults as well as patients with diabetes, diabetic retinopathy, diabetic macular edema, age-related macular degeneration, and retinal vascular occlusion. Most investigations were conducted at single centers, involved relatively small samples, and recruited participants from restricted geographical areas. Differences in age, systemic and ocular disease, nutritional status, and VDD severity therefore limit comparability and generalizability. Moreover, studies that included both eyes did not always clearly account for within-subject inter-eye correlation.

The certainty and comparability of the available evidence are limited by important study-level methodological concerns. Most studies were observational, frequently cross-sectional, and included relatively small or single-center cohorts; therefore, temporal and causal relationships cannot be established. Definitions of VDD varied, particularly regarding serum 25-hydroxyvitamin D thresholds of 20 or 30 ng/mL. Vitamin D measurements may have been influenced by season, sunlight exposure, dietary intake, previous supplementation, geographical latitude, and laboratory assay. Adjustment for potential confounders was inconsistent, particularly for age, sex, body mass index, season and sunlight exposure, diabetes and glycemic control, hypertension, renal function, medication or supplement use, axial length, refractive error, and other ocular characteristics. Furthermore, retinal and choroidal measurements were obtained using different OCT and OCTA devices, scan dimensions, segmentation boundaries, image-quality thresholds, artifact-correction procedures, and proprietary algorithms. Consequently, OCTA vessel density values cannot be directly compared or pooled numerically across platforms or acquisition protocols. Similarly, absolute values for FAZ parameters, choroidal thickness, and CVI cannot be assumed to be interchangeable across studies, and some observed differences may reflect technical rather than biological variation. Although SANRA was used to assess the quality of the present narrative review, a formal design-specific risk-of-bias assessment of each primary study was not performed, which represents an additional limitation. The findings were therefore interpreted qualitatively and cautiously, with greater emphasis placed on consistency of direction than on direct numerical comparisons, and they should not be considered evidence of causality or therapeutic efficacy.

Finally, this narrative review included 23 original studies and did not incorporate a formal risk-of-bias assessment or meta-analysis. Heterogeneity in populations, biochemical definitions, imaging protocols, and reported outcomes made quantitative pooling inappropriate. Accordingly, the findings should be regarded as a qualitative synthesis of emerging evidence rather than a definitive estimate of the magnitude, independence, or clinical significance of the relationship between vitamin D status and ocular microvascular health.

### 9.2. Clinical Implications

The available evidence supports cautious clinical interpretation rather than an immediate change in ocular screening or treatment. OCT and OCTA abnormalities associated with VDD, including reduced SCP or DCP VD, altered FAZ morphology, and lower CT or CVI, are not specific to vitamin D status and may also reflect age, diabetes, hypertension, ocular biometry, or technical imaging factors. OCTA cannot therefore diagnose VDD, and serum 25(OH)D concentration should not be assumed to explain an abnormal ocular vascular measurement independently. Nevertheless, assessment of vitamin D status may be considered as part of a broader systemic evaluation when otherwise unexplained retinal or choroidal abnormalities are identified in patients with clinical risk factors for deficiency.

The detection of vascular changes in otherwise healthy adults and children suggests that OCTA may identify subclinical abnormalities before conventional structural measurements are consistently affected. However, the absence of validated diagnostic thresholds, longitudinal progression data, and established relationships with visual function currently precludes routine OCTA screening of asymptomatic individuals with VDD. In patients with diabetes, age-related macular degeneration, or retinal vascular occlusion, management should continue to prioritize established ocular and systemic risk factors because the independent contribution of VDD remains uncertain.

Functional outcomes are required to determine whether imaging differences have clinical significance. Evidence from neurological disease illustrates this distinction. In 258 patients with progressive multiple sclerosis, serum vitamin D was not significantly associated with peripapillary RNFL thickness, macular volume, GCIPL thickness or low-contrast visual acuity, the corresponding correlations being weak [73]. Similarly, the EVIDIMS sub-analysis compared high-dose with low-dose vitamin D supplementation in 53 patients with relapsing–remitting multiple sclerosis, and while a substantially greater increase in serum vitamin D after 18 months in the high-dose group, no significant differences in pRNFL or GCIPL thickness or in the evaluated brain, brainstem, spinal cord, and thalamic measures were found. A difference in inner nuclear layer thinning was attributable mainly to participants with previous optic neuritis rather than a consistent treatment effect [74]. Observational evidence from demyelinating optic neuritis has associated lower 25(OH)D with prolonged visual evoked potential P100 latency and lower amplitude, but such findings have been observed over time in these patients, with no direct evidence that supplementation improves electrophysiological recovery [75,76].

Vitamin D correction should be individualized according to systemic comorbidities, particularly renal disease. In end-stage renal disease, ocular abnormalities cannot be attributed to VDD alone. Chronic RNFL and ganglion cell layer thinning, reduced macular and peripapillary VD, enlargement of the FAZ and decreased CT in patients with end-stage renal disease may indicate that renal microvascular dysfunction and dialysis could independently influence ocular biomarkers evaluated in VDD [77]. Altered vitamin D metabolism and calcium–phosphate homeostasis also increase the complexity of supplementation, whereas excessive dosing, particularly with calcitriol or other active vitamin D analogue, may promote hypercalcemia, hyperphosphatemia, and vascular or soft-tissue calcification. The potential ophthalmic consequences of severe mineral–vascular dysregulation are illustrated by cases of calciphylaxis without typical cutaneous manifestations [78]. Vitamin D treatment in this population should therefore be medically supervised, with monitoring of serum 25(OH)D, calcium, phosphate, parathyroid hormone, and renal function.

## 10. Conclusions and Future Directions

This narrative review indicates that lower serum vitamin D concentrations are associated with retinal and choroidal vascular alterations across adult and pediatric populations, including variations in SCP and DCP vessel density, retinal vascular caliber and FAZ morphology, as well as lower CT or CVI. However, the direction and magnitude of these associations were not uniform, and conventional retinal structural measurements frequently remained preserved. Although VDR signaling provides biologically plausible links with endothelial function, inflammation, oxidative stress, angiogenesis, barrier integrity, and neurovascular regulation, current evidence does not establish that VDD causes ocular microvascular injury or that vitamin D supplementation provides functional visual benefits. Vitamin D status should therefore be regarded as a potential systemic modifier or biomarker of ocular vascular health rather than an established therapeutic target, and supplementation should continue to follow accepted systemic indications and individual patient requirements.

Prospective longitudinal studies are needed to establish temporality, while randomized controlled trials should determine whether biochemically confirmed vitamin D correction produces sustained OCT/OCTA changes accompanied by improvements in visual acuity, contrast sensitivity, visual fields, or, where relevant, visual evoked potentials. Such studies should use standardized supplementation regimens and harmonized imaging protocols covering scan dimensions, segmentation, artifact correction, and definitions of VD, FAZ morphology, CT, and CVI, while evaluating healthy, pediatric, diabetic, and anti-VEGF-treated populations separately. Automated image analysis may improve quality control, segmentation, and quantitative assessment and could enable integration of ocular imaging with serum vitamin D and systemic biomarkers; however, any VDD-specific predictive models will require development and external validation in large, multicenter, device-diverse datasets before clinical application.

## Figures and Tables

**Table 1:** Studies evaluating vitamin D status and retinal and choroidal imaging findings in adult populations.

Author	Patients	Vitamin D Level ng/mL	Age	Parameters	Results	Key Aspects
Yang et al. 2026 [57]	83 eyes of 83 diabetic patients in 3 groups			SCP FAZ Retinal PD	• G1 vs. G2: Decreased SCP PD in whole, T and S (all rings), and in I (1–3 mm). • G1 vs. G3: Decreased PD in all evaluated SCP regions and rings. • G2 vs. G3: Decreased SCP PD in the whole macular area and S (1–6 and 3–6 mm), N (3–6 mm) • Weak positive correlation between vitamin D levels and SCP PD in most macular regions. • Weak correlation between vitamin D levels and FL PD in the inferior perifoveal region. • Weak correlation between vitamin D levels and FAZ area.	• In VDD patients, PD appeared particularly sensitive to alterations in the temporal perifoveal region.
	G1: serum vitamin D < 20 ng/mL, n = 26	13.9 ± 3.8	48.0 ± 16.3			
	G2: 20 ng/mL ≤ serum vitamin D < 30 ng/mL, n = 30	25 ± 2.7	53.6 ± 12.6			
	G3: normal vitamin D levels, n = 27	36.8 ± 6.2	56.9 ± 13.3			
Tarași et al. 2026 [56]	60 VDD	18.29 ± 5.62	33.25 ± 9.2	FRT GCC SCP DCP FAZ	• Lower FRT • Lower SCP (Inner, Outer) • Lower DCP (whole, inner outer) • Lower GCL thickness in inner nasal inferior • Positive correlation with FRT, DCP)	• VDD is associated with subtle macular and GCC thinning and reduced parafoveal capillary perfusion in otherwise healthy young adults. • OCT and OCTA may detect subclinical retinal alterations before significant visual impairment develops. • VDD could be a factor associated with increased retinal vulnerability and reduced retinal resilience over time.
	vs. 60 age-matched controls	37.89 ± 4.82	33.55 ± 10.01			
Ma et al. 2025 [58]	27 VDD diabetic eyes	15.1 ± 3.4	48.63 ± 15.3	FRT RNFL GC-IPL CT	• Decreased FRT in S and T parafoveal sectors. • Decreased RNFL thickness across all parafoveal sectors. • Decreased GC-IPL thickness in the foveal region and the S, T, N parafoveal sectors. • No significant difference in CT.	• Diabetic patients may present changes independent of VDD. • VDD was associated with accelerated Inner retina layer thinning.
	vs. 29 diabetic eyes without VDD	26.2 ± 4.5	50.3 ± 11.7			
Wei et al. 2025 [9]	286 eyes from 150 diabetic patients grouped into:			FAZ SCP DCP GCL	• Decreased DCP (whole, perifovea), SCP (whole, perifoveal, parafoveal) • Positive correlation between vitamin D and VD in SCP and DCP • Negative correlation between vitamin D and FAZ	• Vitamin D values may be useful in evaluating risk of DR in diabetic patients.
	74 VDD patients	15.8 (14.3–17.8)	56 (48–63)			
	76 without VDD	25.4 (22.3–28.4)	60 (51–66)			
Gürbostan Soysal et al. 2023 [5]	42 eyes from adults with VDD	8.3 ± 3.2	32.17 ± 8.84	CVI SFCT CMT	• Lower CVI • Lower SFCT	• VDD was associated with decreased SCFT and CVI.
	vs. 51 age-matched controls	38.44 ± 3.4	34.31 ± 7.8			
Khater et al. 2025 [8]	68 patients with DME	23.9 ± 3.8	58 ± 7	SCP DCP FAZ FRT	• Positive correlation between vitamin D and SCP (foveal, parafoveal, perifoveal), DCP (foveal, parafoveal and perifoveal) • Negative correlation between vitamin D and diabetes duration, FAZ	• No relation between vitamin D levels and MT. • Vitamin D was able to predict SCP, DCP, FAZ in a multivariate regression model. • DMI was negatively correlated with vitamin D levels.
Kabataș et al. 2022 [59]	114 AMD patients	14.4 ± 9.6	71.5 ± 7.9	FRT CT	• AMD patients had lower CT. • Lower vit D levels were found in patients with subretinal fibrosis compared to without in AMD • Advanced AMD stage presented lower vit D levels.	• FRT did not differ significantly between groups, while CT was lower in AMD patients. • VDD was associated with AMD progression. • Neovascular choroidal diseases may be increased in patients with VDD.
	vs. 102 controls	29.4 ± 14.6	69.4 ± 10.1			
Karabulut et al. 2022 [7]	98 VDD eyes	15.3 ± 4.7	44.3 ± 9.3	SCP DCP CC flow area FAZ	• Increased DCP (whole, perifoveal, parafoveal) • Negative correlation between vitamin D and VD in DCP	• Increased VD may be used to evaluate early microvascular damage.
	vs. 96 healthy age-matched controls	34.6 ± 3.4	48.2 ± 5.3			
Icel et al. 2022 [6]	82 VDD eyes	7.61 ± 3.27	37.29 ± 12.76	FAZ SCP DCP CMT	• Lower SCP, DCP, CMT • Larger FAZ • Positive correlation between vitamin D and SCP and DCP VD. • Negative correlation between vitamin D and FAZ.	• VDD was associated with lower macular perfusion parameters. • FAZ area may be useful in evaluating VDD supplementation efficiency.
	vs. 50 age-matched controls	25.39 ± 4.16	39.1 ± 11.59			
Vural et al. 2021 [60]	30 VDD patients	8.2 ± 2.5	30.5 ± 6.7	CT FRT	• Decreased CT • (subfoveal, nasal, inferior) • Positive correlation between Vitamin D levels and CT (subfoveal, nasal, inferior)	• Inferior region could be naturally thin in healthy eyes, being more subjective to changes. • Structural retinal changes due to VDD may not be detectable at a young age. • Choroidal thinning at a young age is correlated with VDD in relation to vascular endothelial function.
	vs. 30 age-matched controls	28 ± 4.9	28 ± 5.78			

AMD, age-related macular degeneration; CC, choriocapillaris; CMT, central macular thickness; CT, choroidal thickness; CVI, choroidal vascularity index; DCP, deep capillary plexus; DME, diabetic macular edema; DMI, diabetic macular ischemia; DR, diabetic retinopathy; FAZ, foveal avascular zone; FL, full retinal layer; FRT, foveal retinal thickness; GC-IPL, ganglion cell–inner plexiform layer; GCC, ganglion cell complex; GCL, ganglion cell layer; I, inferior; MT, macular thickness; N, nasal; OCT, optical coherence tomography; OCTA, optical coherence tomography angiography; PD, perfusion density; RNFL, retinal nerve fiber layer; S, superior; SCP, superficial capillary plexus; SFCT, subfoveal choroidal thickness; T, temporal; VD, vessel density; VDD, vitamin D deficiency.

**Table 2:** Studies evaluating vitamin D status and retinal and choroidal findings in pediatric populations.

Author	Patients	Vitamin D Level ng/mL	Age	Parameters	Results	Key Aspects
Aydemir et al. 2022 [10]	70 patients with VDD	11.11 ± 4.81	13.14 ± 2.53	CT RNFL FRT, CRAE CRVE	positive correlation between the vitamin D level and the CRAE negatively correlated with the CRVE Lower CT Positive correlation between CT and vitamin D levels	Choroidal thinning could be a potential mechanism in VDD-related retinal diseases. Pediatric findings suggest prolonged vascular alterations in adult retinal diseases with VDD history. Vitamin D supplementation may positively influence the development of retinal diseases.
	vs. 80 age-matched controls	37.26 ± 7.30	13.93 ± 2.93			
Kurtul et al. 2025 [11]	74 eyes of VDD children	11.4 ± 4.9	13.0 ± 3.6	SCP DCP FAZ FA CC FRT RNFL ONH flow	Increased FRT Lower SCP VD (whole, parafoveal, perifoveal) Lower foveal VD in DCP	VDD was associated with increased FRT and lower SCP. Temporal retinal RNFL may be more affected in inflammation due to VDD
	vs. 64 age-matched controls	26.0 ± 11.0	11.7 ± 3.1			
Yüksel Şükün et al. 2025 [61]		Median (IQR)				
	52 malnourished children	13.3 (5.63)	13 (6)	CT CVI	Positive correlation with CVI	CVI showed significant changes in malnourished children. CVI may decrease earlier compared to CT. Vitamin D levels were significantly lower depending on grade of malnutrition.
	vs. 40 age-matched controls	31.9 (8.45)	12.5 (6.5)			
Aksoy Aydemir et al. 2022 [62]	G1: 46 VDD children with type 1 diabetes	12.70 ± 3.22	11.81 ± 3.05	CT RNFL	Decreased CT (subfoveal, nasal, temporal) in G1 vs. G3 and G2 vs. G3. No significant differences in G1 vs. G2. Positive correlation between vitamin D and CT (subfoveal, nasal, temporal) in G1.	VDD was associated with CT thinning in diabetic children. CT thinning in type 1 diabetes without VDD may lead to choroidal ischemia over time.
	G2: 42 children with type 1 diabetes without VDD	29.00 ± 3.20	13.08 ± 3.24			
	G3: 73 controls	30.94 ± 6.24	12.21 ± 2.89			

CC, choriocapillaris; CRAE, central retinal artery equivalent; CRVE, central retinal vein equivalent; CT, choroidal thickness; CVI, choroidal vascularity index; DCP, deep capillary plexus; FAZ, foveal avascular zone; FRT, foveal retinal thickness; ONH, optic nerve head; RNFL, retinal nerve fiber layer; SCP, superficial capillary plexus; T1DM, type 1 diabetes mellitus; VD, vessel density; VDD, vitamin D deficiency.

**Table 3:** Studies of retinal and choroidal changes following vitamin D supplementation.

Author	Patients	Vitamin D Level ng/mL	Age	Parameters	Results	Key Aspects
Karabulut et al. 2025 [65]	72 eyes of 36 VDD patients supplemented with 50,000 IU vitamin D3 weekly for 8 weeks followed by a daily 1200–1500 dose for 4 weeks	Pre- supplementation 12.91 ± 4.15	42.3 ± 7.4	SCP DCP FAZ Flow area	Decreased (whole, parafoveal, perifoveal) VD in DCP in VDD with negative correlation between VD and vit D levels	Retinal microvascular alterations exist after vitamin D supplementation
		After supplementation 24.15 ± 5.48				
	vs. 68 eyes of 34 controls supplemented with 50,000 IU vitamin D3 weekly for 8 weeks followed by a daily 1200–1500 dose for 4 weeks	Pre- supplementation 11.69 ± 3.9	43.2 ± 6.3			
		After supplementation 12.24 ± 5.2				
Fekri et al. 2026 [66]	60 eyes of 30 diabetic patients supplemented with 50,000 IU/week, 8 weeks followed by 800 IU/day	Pre- supplementation 14.20 ± 4.04	58.5 ± 10.2	SCP DCP FAZ	Decreased SCP superior parafoveal	VDD correction had minimal impact on retinal perfusion measured using OCTA. Inflammation and other mechanisms may influence perfusion parameters.
		After supplementation 55.82 ± 6.79				
Kocaay et al. 2023 [63]	83 VDD children received 300,000 IU orally and re-evaluated after 3 months	Pre- supplementation 10.63 ± 5.71	12.3 ± 3.74	CT CVI FRT	Decreased CT and CVI Increased CT and CVI after vitamin D supplementation in VDD patients.	CT and CVI parameters showed an increase in values after supplementation in more severe VDD.
		After supplementation 28.67 ± 5.29				
	vs. 85 controls	27.67 ± 7.26	11.2 ± 4.25			

CC, choriocapillaris; CT, choroidal thickness; CVI, choroidal vascularity index; DCP, deep capillary plexus; FAZ, foveal avascular zone; FRT, foveal retinal thickness; IU, international units; LA, luminal area; OCT, optical coherence tomography; OCTA, optical coherence tomography angiography; SA, stromal area; SCP, superficial capillary plexus; TCA, total choroidal area; VD, vessel density; VDD, vitamin D deficiency.

**Table 4:** Studies evaluating vitamin D status and correction in patients receiving anti-VEGF therapy.

Author	Patients	Vitamin D Level ng/mL	Age	Results	Key Aspects
Fekri et al. 2022 [67]	G1: 29 DME eyes with IVB, without VDD	43.86 ± 6.06	59.97 ± 7.89	DME eyes with VDD showed poorer functional responses to IVB treatment. DME eyes with VDD also showed poorer anatomical responses over six months. Correction of VDD resulted in outcomes comparable to those observed in patients with sufficient vitamin D levels. Significant visual and anatomical superiority was found in G1 and G3 after 6 months.	The unfavorable effect of VDD appeared mainly during later follow-up through DME rebound or interruption of gradual improvement. VDD correction could provide better and more stable long-term results in DME eyes under IVB treatment.
	G2: 26 DME eyes with VDD + IVB	17.38 ± 4.59	59.46 ± 8.76		
	G3: 28 eyes with VDD + IVB + vit D supplementation (50,000 IU/week, 8 weeks, followed by 800 IU/day)	16.61 ± 4.35 post sup: 41.68 ± 6.09	59.57 ± 7.00		
Karimi et al. 2022 [68]	70 eyes of 68 patients with 3-monthly IVB with A: CRVO, B: BRVO grouped into:			No correlation between vit D levels and BCVA or CMT at baseline. CMT was significantly decreased in all BRVO groups following 3-monthly IVB injections. No difference between groups. CMT was significantly decreased in all CRVO groups following 3-monthly IVB injections. VDD levels significantly influenced CMT recovery.	VDD correction positively influenced BCVA and CMT in CRVO patients with IVB treatment compared to controls.
	G1: without VDD	G1A: 40.31 ± 12.74 G1B: 40.68 ± 15.76	G1A: 60.5 ± 7.81 G1B: 63.41 ± 6.94		
	G2: VDD with vit D supplementation (50,000 IU/week, 8 weeks)	G2A: 18.3 ± 7.83 G2B: 23.65 ± 4.72	G2A: 56.57 ± 5.06 G2B: 65.5 ± 5.49		
	G3: VDD	G3A: 19.49 ± 6.32 G3B: 18.07 ± 8.49	G3A: 58.38 ± 9.69 G3B: 60.92 ± 9.81		
Karimi et al. 2021 [69]	71 DME patients with 3-monthly IVB grouped into:			Negative correlation between HbA1c and serum vitamin D. CMT decreased in all 3 groups without statistically significant difference between them after 3-monthly IVB injections.	VDD correction had a positive effect on CMT reduction without a statistically significant difference.
	G1: 37 without VDD	36.5 ± 6.7	65 ± 6		
	G2: 19 VDD with supplementation 5000 IU/week for 8 weeks	17.3 ± 5.4	64 ± 9		
	G3: 15 VDD without supplementation	20.3 ± 5	59 ± 8		

BCVA, best-corrected visual acuity; BRVO, branch retinal vein occlusion; CDVA, corrected distance visual acuity; CMT, central macular thickness; CRVO, central retinal vein occlusion; DME, diabetic macular edema; IVB, intravitreal bevacizumab; IU, international units; RVO, retinal vein occlusion; VEGF, vascular endothelial growth factor; VDD, vitamin D deficiency.

**Table 5:** Studies evaluating serum vitamin D status in retinal vascular occlusions.

Author	Patients	Vitamin D Level ng/mL	Age	Results	Key Aspects
Kandambeth et al. 2023 [70]	35 RVO patients	7 RVO < 20 ng/mL and 20 ng/mL < 28 RVO < 30 ng/mL	60.09 ± 10.84	All RVO patients had Vitamin D < 30 ng/mL	Vitamin D may be a risk factor in the causal mechanism of RVO.
	35 controls	20 ng/mL < 10 control < 30 ng/mL and 30 ng/mL < 25 control	60.2 ± 10.86		
Bhanot et al. 2024 [71]	50 patients grouped by CRVO/BRVO/CRAO/BRAO regrouped by:			Negative correlation between FRT and Vitamin D levels in RVO. Significant Vitamin D difference between ischemic and non-ischemic subgroups of vascular occlusions.	VDD may be involved in the spectrum of retinal vascular diseases. Vitamin D supplementation could be considered as a target therapy, especially in at-risk patients for vascular ischemic disorders.
	ischemic	18.85 ± 3.11	62 ± 10.4		
	nonischemic	19.11 ± 2.46			
	50 controls	32.69 ± 11.94	60 ± 9.7		
Bhattacharya et al. 2025 [72]	50 RVO patients	21.82 ± 9.28	46.36 ± 8.6	low levels of Vitamin D were found in the cases of RVO compared to controls. No difference between RVO subgroups.	VDD may be a factor in developing RVO.
	vs. 50 controls	30.71 ± 6.85	44.56 ± 9.57		

BRAO, branch retinal artery occlusion; BRVO, branch retinal vein occlusion; CRAO, central retinal artery occlusion; CRVO, central retinal vein occlusion; FRT, foveal retinal thickness; RVO, retinal vein occlusion; VDD, vitamin D deficiency.

## Data Availability

The original contributions presented in this study are included in the article. Further inquiries can be directed to the corresponding author.

## Abbreviations

The following abbreviations are used in this manuscript:

25(OH)D	25-Hydroxyvitamin D
AMD	Age-related macular degeneration
BCVA	Best-corrected visual acuity
BRAO	Branch retinal artery occlusion
BRVO	Branch retinal vein occlusion
CC	Choriocapillaris
CDVA	Corrected distance visual acuity
CMT	Central macular thickness
CRAE	Central retinal artery equivalent
CRAO	Central retinal artery occlusion
CRVE	Central retinal vein equivalent
CRVO	Central retinal vein occlusion
CT	Choroidal thickness
CVI	Choroidal vascularity index
DCP	Deep capillary plexus
DME	Diabetic macular edema
DMI	Diabetic macular ischemia
DR	Diabetic retinopathy
FAZ	Foveal avascular zone
FL	Full retinal layer
FRT	Foveal retinal thickness
GCC	Ganglion cell complex
GC–IPL	Ganglion cell–inner plexiform layer
GCL	Ganglion cell layer
IVB	Intravitreal bevacizumab
LA	Luminal area
OCT	Optical coherence tomography
OCTA	Optical coherence tomography angiography
ONH	Optic nerve head
PD	Perfusion density
RNFL	Retinal nerve fiber layer
RPE	Retinal pigment epithelium
RVO	Retinal vein occlusion
SA	Stromal area
SCP	Superficial capillary plexus
SFCT	Subfoveal choroidal thickness
T1DM	Type 1 diabetes mellitus
TCA	Total choroidal area
VD	Vessel density
VDD	Vitamin D deficiency
VDR	Vitamin D receptor
VEGF	Vascular endothelial growth factor

## Appendix A. Database-Specific Search Approach and Search Terms

The literature search consisted of a primary structured PubMed search supplemented by targeted searches in Scopus and ScienceDirect. The primary PubMed search was conducted on 5 July 2026 and covered studies published between 2021 and 2026. Separate searches were performed using combinations of terms relevant to vitamin D status, retinal and choroidal vascularization, and OCT/OCTA imaging. The supplementary searches in Scopus and ScienceDirect were adapted to the specific topic being investigated and therefore did not necessarily apply all search terms or filters simultaneously. Eligibility was restricted to English-language original human studies evaluating vitamin D status or supplementation in relation to retinal or choroidal vascular outcomes. Records identified through the primary and supplementary searches were combined before eligibility assessment and duplicate removal.

**Database**	**Search Approach**	**Principal Search Terms and Combinations Used**
PubMed	Primary structured literature search	“vitamin D”; “retina”; “retinal”; “vascular”; “optical coherence tomography”; “optical coherence tomography angiography”; “OCT”; “OCTA”. Separate combinations included “vitamin D” AND “retina” AND “vascular”, “vitamin D” AND “optical coherence tomography”, and “vitamin D” AND “optical coherence tomography angiography”.
Scopus	Supplementary targeted search	Combinations of “vitamin D” with terms related to the retina or retinal/choroidal vascularization, including “retina”, “retinal”, “vascular”, “optical coherence tomography”, “optical coherence tomography angiography”, “OCT”, and “OCTA”. Searches were adapted according to the specific topic being investigated.
ScienceDirect	Supplementary targeted search	Combinations of “vitamin D” with terms related to the retina or retinal/choroidal vascularization, including “retina”, “retinal”, “vascular”, “optical coherence tomography”, “optical coherence tomography angiography”, “OCT”, and “OCTA”. Searches were adapted according to the specific topic being investigated.

## Appendix B. SANRA Checklist Appraisal

The methodological quality of the narrative review was appraised using the Scale for the Assessment of Narrative Review Articles (SANRA). Each of the six domains was scored from 0 to 2, with a maximum total score of 12.

**SANRA Domain**	**Score (0–2)**	**Rationale**
(1) Justification of the article’s importance	2	The Introduction establishes the biological and clinical relevance of vitamin D to retinal and choroidal microvascular health and identifies a fragmented evidence base across populations, imaging protocols, ocular diseases, and therapeutic settings, supporting the need for an integrated narrative synthesis.
(2) Statement of aims	2	The aim is explicitly stated at the end of the Introduction and encompasses OCTA findings in adult and pediatric populations, vascular changes following vitamin D supplementation, the influence of VDD and its correction on anti-VEGF treatment response, and retinal vascular occlusive diseases.
(3) Description of the literature search	2	The Methods describe the primary structured PubMed search and supplementary targeted searches in Scopus and ScienceDirect, including the search period, principal search concepts, eligibility criteria, screening process, data extraction, and synthesis approach. The database-specific search approach and principal search terms are provided in Appendix A.
(4) Referencing	2	Relevant statements concerning biological mechanisms, imaging findings, supplementation, anti-VEGF therapy, and retinal vascular occlusive diseases are supported by appropriate references, with primary clinical studies cited when their findings are discussed and compared.
(5) Scientific reasoning	2	The review critically evaluates inconsistent findings, methodological heterogeneity, potential confounding, study-design limitations, and alternative interpretations. Associations are distinguished from causality, and therapeutic implications are interpreted cautiously according to the strength and limitations of the available evidence.
(6) Appropriate presentation of data	2	Study characteristics and relevant outcomes are presented in thematic tables, including study populations, vitamin D concentrations, age, investigated retinal or choroidal parameters, principal findings, and interpretative aspects. Standardized terminology and abbreviations facilitate comparison across studies.
